# Comparison of allergy prevalence using brinzolamide 1.0% / brimonidine 0.2% fixed combination with and without β-blocker in glaucoma patients: a retrospective cohort study

**DOI:** 10.1186/s12886-024-03550-2

**Published:** 2024-07-11

**Authors:** In Ki Park, Seon Ha Bae, Jae Hoon Jeong, Kyoung Woo Kim, Kayoung Yi, Yeoun Sook Chun

**Affiliations:** 1 Department of Ophthalmology, Kyung Hee University College of Medicine, Kyung Hee University Hospital, Seoul, Republic of Korea; 2 Department of Ophthalmology, Chung-Ang University Hospital, Chung-Ang University College of Medicine, Seoul, Republic of Korea; 3 Department of Ophthalmology, Chung-Ang University College of Medicine, Chung-Ang University Gwangmyeong Hospital, Gwangmyeong City, Gyeonggido Republic of Korea; 4 Department of Ophthalmology, Hallym University College of Medicine, Hallym University Kangnam Sacred Heart Hospital, Seoul, Republic of Korea

**Keywords:** Allergy, Blepharoconjunctivitis, Brinzolamide 1.0%/brimonidine 0.2% fixed combination, Beta blocker, Papillary conjunctivitis, Follicular conjunctivitis

## Abstract

**Background:**

Glaucoma treatment often involves multi-drug regimens, which can lead to poor adherence and side effects. Fixed-dose combinations aim to improve adherence and reduce side effects compared to traditional therapies. This study aimed to compare the prevalence and clinical characteristics of ocular allergy in glaucoma patients using brinzolamide 1.0%/brimonidine 0.2% fixed combination (BBFC), with and without concurrent β-blocker.

**Methods:**

Of these, 176 patients used a β-blocker concurrently, whereas 96 patients did not. Allergy prevalence, allergy type, and allergy occurrence time were compared between the concurrent and non-concurrent β-blocker-usage groups. Ocular allergies were classified and evaluated using Kaplan–Meier survival analysis.

**Results:**

Allergy prevalence was 10.23% and 15.63% (*p* = 0.193), whereas allergy occurrence time was 15.92 ± 13.80 months and 6.26 ± 6.20 months (*p* = 0.04) in the concurrent and non-concurrent β-blocker-usage groups, respectively. Kaplan–Meier survival analysis indicated that half of the allergies in the concurrent β-blocker-usage group occurred within 12.5 months, with the BBFC discontinuation rate gradually increasing up to 36 months. Contrarily, half of the allergies in the non-concurrent β-blocker-usage group occurred within 3.3 months, with a rapid increase in BBFC discontinuation rate the first 6 months. Intergroup differences in allergy types were significant (*p* = 0.015). Among all patients with allergy, the average allergy occurrence time of blepharoconjunctivitis, papillary conjunctivitis, and follicular conjunctivitis was 12.52, 9.53, and 13.23 months, respectively. Follicular conjunctivitis tended to occur later than papillary conjunctivitis (*p* = 0.042). In the concurrent β-blocker-usage group, follicular conjunctivitis was the most prevalent allergy type (61.1%), whereas papillary conjunctivitis was the most common (66.7%) in in the non-concurrent β-blocker-usage group.

**Conclusions:**

Concurrent use of β-blocker with BBFC decreases allergy prevalence, delays allergy onset, and predominantly results in follicular conjunctivitis, thereby facilitating longer treatment duration. Understanding these characteristics of allergy in BBFC users is useful to manage patients and improve treatment adherence. This study provides insights into the role of β-blockers in modulating ocular allergy in BBFC-treated glaucoma patients, highlighting implications for clinical practice and patient education.

## Background

Glaucoma is a chronic and progressive optic neuropathy for which the long-term lowering of the intraocular pressure (IOP) remains the only proven treatment. [1, 2] Kass et al. [3] reported that approximately 40% of the patients with glaucoma need at least two and 9% need more than three eyedrops to reach the target IOP. However, the prescription of multiple drugs increases the risk of poor patient adherence and higher exposure to the preservatives that are contained in eyedrops, which can cause various side effects. Fixed-dose combinations, wherein two different components are formulated together in a single container, have been developed and have widespread usage. The advantages of fixed-dose combinations included improved adherence because of the simplified dosing schedule, prevention of the possible dilution of the first drug by the second drug, and lower preservative exposure of the ocular surface. [4, 5]

The majority of the fixed-dose combination formulations include β-adrenergic antagonists, such as timolol 0.5%, which has limited applicability for patients with local allergies or systemic diseases, including chronic obstructive pulmonary disease, asthma, cardiovascular disease, and sinus bradycardia. [6] The 1.0% brinzolamide–0.2% brimonidine fixed-dose combination (BBFC), which was approved by the US Food and Drug Administration in 2013, is the only fixed-dose combination that does not contain timolol and therefore may be used by patients with restrictions on β-blocker use. [7] Furthermore, BBFC can be used with prostaglandins and the timolol fixed-dose combinations when marked IOP reduction is required as it enables the application of four drugs from two bottles into the eye.

Drug-induced allergy is an important side effect that decreases patient adherence. Despite the high prevalence of ocular allergy, the widespread application of brimonidine in the treatment of glaucoma is attributable to its ability to effectively decrease the IOP while supporting neuroprotection and only rare cardiovascular side effects in adults. [8–10] Brimonidine-induced allergy has a prevalence of 3.5–22.02%, depending on the concentration, number of eyedrops instilled, and the characteristics of the drug components of the combined-drug formulation. [11–15]

A fixed-dose combination containing 0.2% brimonidine (0.2% brimonidine–0.5% timolol fixed-dose combination, BTFC; Combigan^®^, Allergan Inc., Irvine, CA, USA) was, despite its higher concentration, associated with a 50% lower prevalence of allergy than a single-drug lower-concentration formulation (0.15% brimonidine; Alphagan-P^®^, Allergan Inc., Irvine, CA, USA). [13] Similarly, BTFC has a 50% lower prevalence of ocular allergy than 0.2% brimonidine monotherapy. [15] The lower prevalence of allergy with BTFC is attributable to the timolol-mediated inhibition of the brimonidine-induced allergy. [16, 17]

The 0.2% brimonidine concentration in BBFC could induce considerable allergic reactions. However, unlike that for other glaucoma drugs, research into BBFC-related side effects is limited. Ascertaining the role of β-blockers in BBFC-induced ocular allergy could aid the selection of glaucoma treatment as well as enable patient education and counseling in the real-world clinical setting.

This study was conducted to compare the prevalence, onset timing and characteristics of BBFC-related ocular allergy in relation to concurrent β-blocker usage or non-usage in order to evaluate the role of β-blockers in dampening ocular allergy.

## Methods

In this retrospective study, we reviewed the medical records of 286 glaucoma patients, who used Simbrinza^®^ (Alcon, Inc., Fort Worth, TX, USA) twice a day, at two institutions from March 2016 to November 2021. The study was conducted in accordance with the principles underlying the Declaration of Helsinki and was approved by the institutional review board (IRB No. 2002-008-19302, HKS 2021-01-0142201-004-19400) of each study center. The requirement for informed consent was waived due to the retrospective nature of the study.

**Fig. 1:**
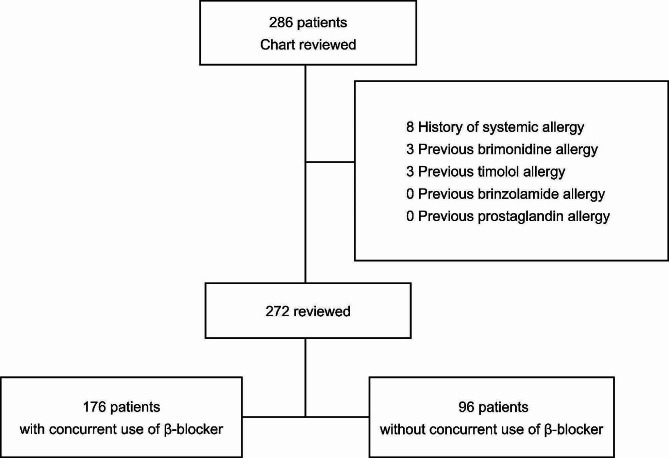
Flowchart of patient screening and participant selection and disposition. Based on the study eligibility criteria, 272 of the 286 patients with glaucoma who were treated with the brinzolamide 1.0%–brimonidine 0.2% fixed-dose combination (BBFC) were enrolled in this study

Among the patients who were screened, eight patients with a history of allergic conjunctivitis, rhinitis, and systemic allergies, including asthma, atopic dermatitis, and hypersensitivity to contrast agents and six patients with a history of ocular allergies to ophthalmic pharmacotherapeutic agents for glaucoma, such as brimonidine, brinzolamide, timolol, and prostaglandin, were excluded from the study (Fig. 1). The remaining 272 participants were categorised into two groups based on concomitant β-blocker usage. The patient’s sex, age, glaucoma type, follow-up period after BBFC treatment initiation, occurrence of allergy, time of onset, and clinical characteristics of allergy were analysed and compared between the two groups: concurrent and non-concurrent β-blocker-usage groups. Glaucoma was classified as primary open angle glaucoma, normal tension glaucoma, primary angle closure glaucoma, and secondary glaucoma according to guideline of the European Glaucoma Society. [18]

Ocular allergy was defined as lid oedema, erythema, itching, and papillary or follicular conjunctivitis accompanied by injection that necessitated discontinuation of BBFC treatment. [11] The follow-up period was calculated from the date that BBFC treatment was initiated to the date of the last visit. The allergy occurrence time was calculated from the date when BBFC treatment was started to the date when BBFC was discontinued after confirmation of the allergy.

Symptoms such as dry mouth, drowsiness, and dizziness were defined as systemic side effects of BBFC, although participants with these symptoms were not included in the group with allergy if there were no symptoms of ocular allergy. Patients with eye discomfort, dry eye syndrome, or irritation were not included in the allergy group. Allergies were classified into three categories: follicular conjunctivitis, papillary conjunctivitis, and blepharoconjunctivitis. Allergy types and allergy occurrence time were compared between the two groups.

Intergroup differences in ocular allergy were evaluated using Kaplan–Meier survival curves. The chi-square or Fisher’s exact test was used for nominal variables, whereas the Mann–Whitney *U* test was used for numerical variables. To evaluate the effect of concurrent β-blocker usage, hazard ratio was calculated for all allergy types, specifically for papillary conjunctivitis. Statistical analyses were performed using IBM SPSS ver. 20 (IBM Corp., Chicago, IL, USA), with a *p*-value < 0.05 considered statistically significant.

## Results

Of the 272 participants receiving BBFC for the treatment of glaucoma, 176 (64.7%) and 96 (35.3%) did and did not use β-blockers concurrently, respectively. Timolol maleate was the only β-blocker used, with 171 participants receiving prostaglandin analogue (PGA)–timolol fixed-dose combinations and 5 participants using 0.5% timolol-only formulations. Meanwhile, 68 participants were BBFC-only users and 28 were PGA–BBFC users. No significant intergroup differences were observed in the mean age, follow-up time, and glaucoma type. The proportion of men was two-fold that of women in the concurrent β-blocker usage group, whereas sex distribution was equal in the non-concurrent β-blocker usage group (Table 1).

**Table 1:** Baseline characteristics of participants using the brinzolamide 1.0%–brimonidine 0.2% fixed-dose combination (BBFC)

	Total	With β-blocker usage	No β-blocker usage	*p*-value
Number of patients (*n*, %)	272 (100)	176 (64.7)	96 (35.3)	-
Mean age (years)	65.72 ± 14.65	64.84 ± 15.19	68.10 ± 13.34	0.120^†^
Sex (male/female)	165/107	120/56	45/51	0.001*
Follow-up time (months)	17.68 ± 16.00 (0.07–53.43)	19.20 ± 16.39 (0.07–53.43)	14.89 ± 14.94 (0.46–51.43)	0.092^†^
Type of glaucoma (*n*, %)				
Primary open-angle	127 (46.7)	73 (41.5)	54 (56.3)	0.061^*^
Normal tension	87 (32.0)	65 (36.9)	22 (22.9)	
Primary closed-angle	22 (8.1)	13 (7.4)	9 (9.4)	
Secondary	36 (13.2)	25 (14.2)	11 (11.5)	
Concurrent topical medication (*n*)				
None			68	
PGAs			28	
β-blocker		5		
FC PGA–β-blocker		171		

Values are presented as the mean ± standard deviation (range) or frequency (percentage)

^†^*p*-value by the Mann–Whitney *U* test; significance set at < 0.05

*p-value by the chi-square or Fisher’s exact test for two-by-two tables; significance set at < 0.05

PGAs: prostaglandin analogues, FC PGA–β-blocker: Fixed-dose combination of PGAs and β-blocker

Table 2 summarises the characteristics of ocular allergy and systemic adverse reactions. Allergy prevalence was 10.23% (18/176) and 15.63% (15/96) in the concurrent and non-concurrent β-blocker usage groups, respectively. Although allergy prevalence was lower in the concurrent β-blocker usage group than in the non-concurrent β-blocker usage group, it was not statistically significant (*p* = 0.193). The average allergy occurrence time was 15.92 ± 13.80 (range: 0.93–45.26) and 6.26 ± 6.20 (range: 0.46–24.73) months in the concurrent and non-concurrent β-blocker usage groups, respectively, with a significantly delayed allergy occurrence (*p* = 0.04) in the concurrent β-blocker usage group.

**Table 2:** Ocular allergic reactions and systemic adverse events

	Total	With β-blocker usage	No β-blocker usage	*p*-value
Number of patients with allergy (%)	33 (12.13)	18 (10.23)	15 (15.63)	0.193*
Onset of allergy (months) (range)	11.53 ± 11.91 (0.46–45.26)	15.92 ± 13.80 (0.93–45.26)	6.26 ± 6.20 (0.46–24.73)	0.040^†^
Type of ocular allergy (*n*)				
Follicular conjunctivitis	13	11	2	0.015*
Papillary conjunctivitis	14	4	10	
Blepharoconjunctivitis	6	3	3	
Systemic adverse event (*n*)	5	4	1	0.659*

Values are presented as the mean ± standard deviation (range) or frequency (percentage)

^†^*p*-value by the Mann–Whitney *U* test; significance set at < 0.05

*p-value by the chi-square or Fisher’s exact test for two-by-two tables; significance set at < 0.05

Significant intergroup differences were observed in the prevalence of three types of ocular allergy (*p* = 0.015) (Table 2). The calculated hazard ratio of β-blocker usage was 0.65 (95% confidence interval, 0.11–5.96) for overall allergy and 0.22 (95% confidence interval, 0.08–0.67) for papillary conjunctivitis. In all patients, the average occurrence time of blepharoconjunctivitis, papillary conjunctivitis, and follicular conjunctivitis was 12.52 ± 5.44, 9.53 ± 2.92, and 13.23 ± 3.58 months, respectively. Follicular conjunctivitis tended to occur later than papillary conjunctivitis (*p* = 0.042). Follicular conjunctivitis (61.1%, 11/18) was predominant in the concurrent β-blocker usage group, whereas papillary conjunctivitis (66.7%, 10/15) was more common in the non-concurrent β-blocker usage. No significant intergroup differences (*p* = 0.659) were observed in the occurrence of systemic adverse reactions.

**Fig. 2:**
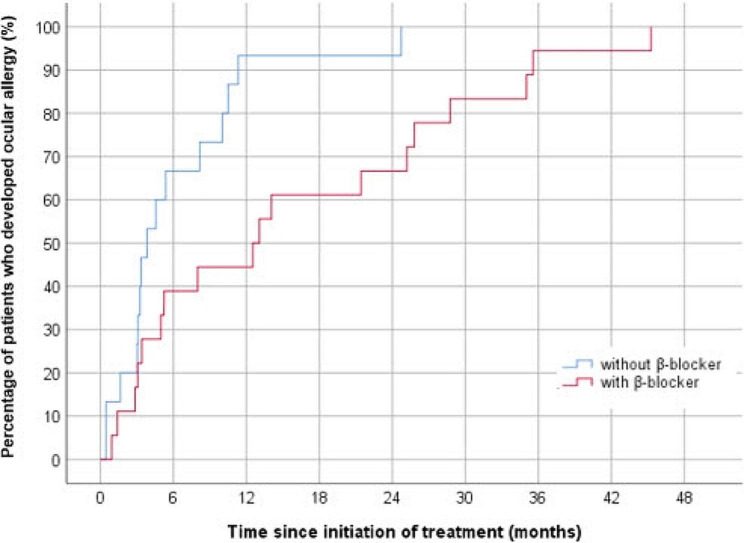
Kaplan–Meier survival curve of the occurrence of ocular allergy in patients treated with the 1.0% brinzolamide–0.2% brimonidine fixed-dose combination (BBFC), with or without concurrent β-blocker usage. Half of the allergic reactions occurred after 12.5 and 3.3 months in the concurrent β-blocker usage and no-concurrent β-blocker usage groups, respectively. Compared to the no-concurrent β-blocker usage group, the concurrent β-blocker usage group had slower onset of allergy

**Fig. 3:**
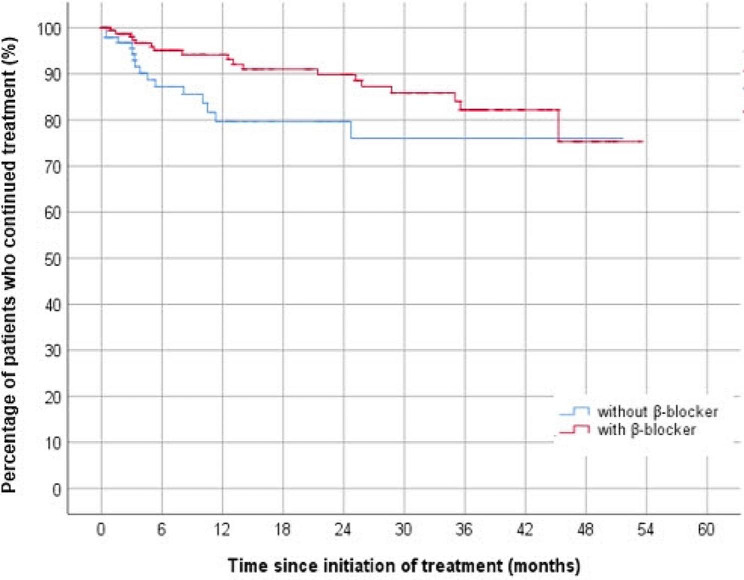
Kaplan–Meier survival curve of the comparison of the rate of discontinuation of the 1.0% brinzolamide–0.2% brimonidine fixed-dose combination, (BBFC) with or without concurrent β-blocker usage. In the group with concurrent β-blocker usage, the rate of discontinuation of BBFC gradually increased to 36 months. In contrast, in the no-concurrent β-blocker usage group, the rate of discontinuation of BBFC increased rapidly during the first 6 six months, and the majority of patients discontinued BBFC treatment within 12 months

Kaplan–Meier survival analysis of allergy prevalence of BBFC revealed a significant difference between the two groups. Half of the allergic reactions occurred after 12.5 months in the concurrent β-blocker usage group and before 3.3 months in the non-concurrent β-blocker usage group. The concurrent β-blocker usage group exhibited slower allergy onset than the non-concurrent β-blocker usage group (Fig. 2). Allergy-induced BBFC discontinuation in the concurrent β-blocker usage group increased gradually over 36 months, whereas it increased rapidly during the first 6 months and peaked within 12 months in the concurrent β-blocker usage group (Fig. 3).

## Discussion

BBFC, which contains 0.2% brimonidine and 1% brinzolamide, is the only fixed-dose combination of eyedrops devoid of timolol maleate. Brinzolamide noncompetitively inhibits carbonic anhydrase II in the ciliary epithelium, thereby decreasing the formation of bicarbonate ions, inhibiting the transportation of sodium and fluid across the ciliary epithelium, which results in decreased production of aqueous humor. [19] Brimonidine selectively acts on α_2_-adrenergic receptor to decrease aqueous production via the constriction of iridial and ciliary vessels and the inhibition of adenyl cyclase. Furthermore, by enhancing prostaglandin release, brimonidine increases uveoscleral outflow [20, 21] and may have a neuroprotective effect on ganglionic cell axons. [22] Brimonidine reduces the volume of conjunctival epithelial cells and consequently widens intercellular spaces, potentially triggering an allergic reaction through the facilitated movement of proinflammatory substances through widened intercellular spaces into the subconjunctival tissue. [16] BBFC is rapidly metabolised by cytochrome P450, minimising the effect on cardiovascular and pulmonary functions. [23] Therefore, BBFC can be used safely in patients with hypotension, bradycardia, asthma, and chronic obstructive pulmonary disease, which may be adversely affected by the systemic side effects of β-blockers. [6] In cases requiring significant IOP reduction, a prostaglandin–timolol fixed-dose formulation can be combined effectively with BBFC to ensure fewer number of eyedrops.

The lower allergy rate of the 0.2% brimonidine–0.5% timolol fixed combination than of the brimonidine-only formulation, irrespective of the concentration, has been attributed to β-blockers. [13, 15, 24] However, it is unclear how timolol modulates allergies induced by BBFC containing 0.2% brimonidine.

In the current study, allergy prevalence induced by BBFC was 10.23% with timolol and 15.63% without timolol, suggesting that concomitant β-blocker usage decreased allergy prevalence, although no significant difference was observed. However, clinically there were significant differences. The use of β-blocker lowered the prevalence of papillary conjunctivitis by 0.22 times. The concurrent β-blocker usage group had slower allergy onset (mean duration 15.92 months), which predominantly comprised follicular conjunctivitis, with a gradual increase in allergy prevalence over 36 months. Conversely, the non-concurrent β-blocker usage group had a significantly faster allergy onset (mean duration 6.26 months), which predominantly comprised papillary conjunctivitis, with BBFC discontinuation within 12 months.

**Fig. 4:**
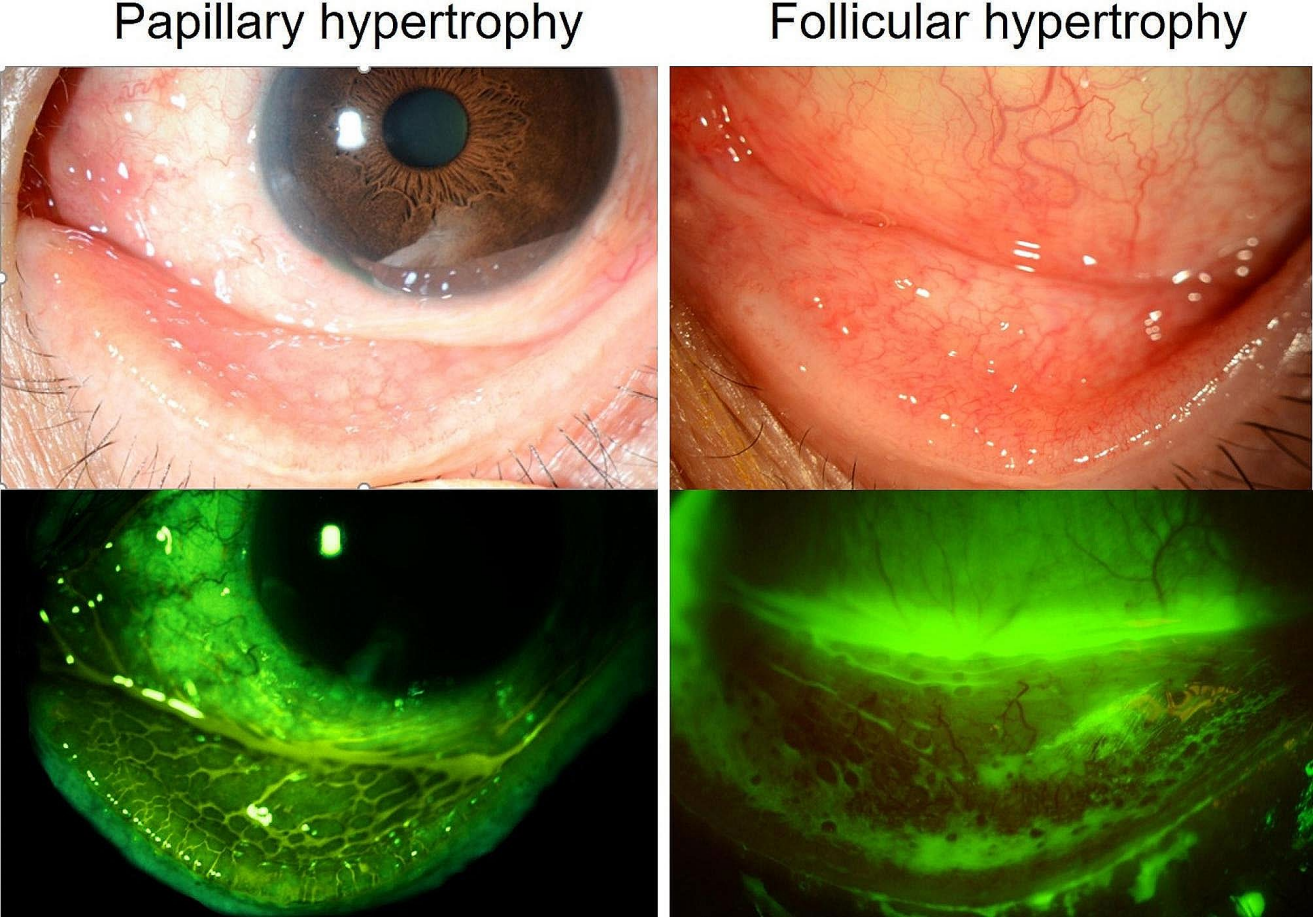
The papillary conjunctivitis seen in the concurrent use of brimonidine and β-blocker group appears as the pale giant papillae induced by prolonged extravasated exudation. The follicular conjunctivitis is characterized by yellow, opaque nodules with aggregated immune cells induced by brimonidine. Fluorescein staining makes it easier and clearer to differentiate papillae and follicle

Brimonidine-induced allergic conjunctivitis presents as papillary and follicular conjunctivitis. Papillary conjunctivitis is caused by a type 1 hypersensitivity reaction, characterised by enlarged papillae with dilated blood vessels and increased vascular permeability due to cytokines secreted from antigen-sensitised mast cells. Brimonidine itself does not act as an antigen but reduces the volume of conjunctival epithelial cells, widening the intercellular spaces, and facilitating the entry of antigens and potential inflammatory substances into subconjunctival tissue, triggering an allergic reaction. [16] β-blockers prevent excessive influx of antigens through inhibition of intracellular volume reduction caused by brimonidine. [25] In addition, β-blockers can induce vasoconstriction. [26] This dual action can reduces antigen influx to mast cells. Moreover, even if the antigen-stimulated mast cells release histamine, timolol can decrease vascular permeability and secondarily inhibit excessive spread of cytokines. We believe that the concurrent timolol usage group exhibited a relatively low prevalence of papillary conjunctivitis and high prevalence of follicular conjunctivitis due to the involvement of these β-blockers. Unlike the non-concurrent β-blocker usage group that demonstrated a rapid-onset type 1 hypersensitivity reaction, the papillary conjunctivitis observed in the concurrent β-blocker usage group revealed pale giant papillae formed by the disruption of the subconjunctival septum due to prolonged extravasated exudation (Fig. 4). The pathogenesis of brimonidine-induced follicular conjunctivitis is attributed to the proliferation and activation of normally resident immune cells in the conjunctiva, stimulated by the lymphoproliferative effects of brimonidine. [27] This condition is less associated with vascular permeability and occurs more slowly than typical allergic reactions, as immune cell proliferation is a time-dependent process and progresses slowly. These conjunctival follicles, visible with fluorescein staining (Fig. 4), are characterised by yellow, opaque nodules with aggregated immune cells and can range from small to giant follicles larger than 1.0 mm in diameter. Rarely, conjunctival thickening due to chronic proliferation of T and B lymphocytes mimic mucosa-associated lymphoid tissue lymphoma. [27, 28] Moreover, brimonidine has been reported to induce granulomatous anterior uveitis. [29] Thus, β-blockers are believed to suppress papillary conjunctivitis manifesting early in allergic reaction, while exerting no effect on the development of follicular conjunctivitis, which emerges later dur to lymphocyte proliferation.

Both types of conjunctivitis typically resolve upon discontinuation of BBFC and do not require any special treatment. Papillary conjunctivitis presents with typical allergic symptoms of redness, itching, discharge, and foreign body sensation and can be controlled with anti-allergic medications. In contrast, follicular conjunctivitis is characterised by milder symptoms, usually itching and discharge, with less redness, and tends to be less responsive to anti-allergic medications, due to the predominant role of follicular proliferation. Therefore, clinicians should differentiate between these two types when treating patients.

The prevalence of allergy to brimonidine is primarily influenced by the number of instillations and concentration of the agent. [12, 14] In this study, the number of instillations and concentration of twice-daily instillations of BBFC containing 0.2% brimonidine was the same as that reported by Sherwood et al. [30] and Motolko [15] using a fixed combination brimonidine 0.2%-timolol 0.5%. However, the prevalence of ocular allergy was 10.23% in the concurrent β-blocker usage group, which was higher than the prevalence reported by Sherwood et al. [30] and Motolko [15] (5.2% and 8.8%, respectively). This difference can be attributed to several factors, including the presence of a fixed combination, duration of treatment, and racial differences. First, previous studies used fixed-dose combinations, whereas our study used β-blockers concurrently. [24] Second, previous studies were prospective for 12–18 months, our study had a longer follow-up period of up to 53 months, potentially increasing the allergy prevalence by including ocular allergies that occurred after 12–18 months. Finally, unlike previous reports wherein the study population was predominantly Caucasian, all the patients in our study were Korean, suggesting plausible racial differences in the susceptibility to brimonidine-induced allergies. Furthermore, brimonidine metabolism is known to be affected by iris pigmentation, [31, 32] as evidenced in animal studies where brimonidine metabolism was slower with higher concentration and affinity in the eyes of rats with darkly pigmented irises than that noted in rats with non-pigmented irises. [33] While these animal experiments cannot directly explain our hypothesis, they cautiously suggest that the relatively higher anterior segment concentrations of brimonidine in Koreans with dark brown irises may contribute to the higher prevalence of allergy.

The study has some limitations. First, the retrospective design and inclusion of patients who discontinued BBFC due to allergy led to unequal follow-up periods between groups. This discrepancy could potentially influence the observed allergy prevalence, as evidenced by the Kaplan–Meier survival curves showing trends over longer durations. If the follow-up period had been the same between two groups, it is possible that the prevalence of allergy may have shown a statistically difference. Second, there is a possibility of selection bias. Majority of the patients (171/176) in the concurrent β-blocker usage group received timolol as part of the fixed combination with PGA. Although the influence of topical timolol is the most probable factor for reduction in allergy rates, the possible interference of the concomitant use of PGA should be considered. Future studies comparing BBFC vs. BBFC + timolol or BBFC + PGA vs. BBFC + PGA + timolol are warranted to provide clearer insights for drawing conclusions. Furthermore, although we excluded patients with known allergies to glaucoma medications were excluded during screening, we cannot entirely rule out allergies to other concomitant ophthalmic medications used by the participants. However, we believe this is unlikely.

## Conclusions

When a β-blocker is used concurrently with BBFC, allergy prevalence is reduced possibly owing to the inhibitory effect of antigen influx and vasoconstriction. Concurrent use of β-blocker can delay the onset of allergy and thereby extend the duration of usage. When using BBFC, understanding the prevalence and timing of allergy, between papillary and follicular conjunctivitis, and comprehending the pathogenesis of each form will be helpful to manage patients with glaucoma, enabling prolonged and safer use of BBFCs.

## Data Availability

The datasets used and/or analysed during the current study are available from the corresponding author on reasonable request.

## Abbreviations

IOP: Intraocular pressure
BBFC: Brinzolamide 1.0%/brimonidine 0.2% fixed combination
PGA: Prostaglandin analogue
